# Immunomodulation—A Molecular Solution to Treating Patients with Severe Bladder Pain Syndrome?

**DOI:** 10.1016/j.euros.2021.07.003

**Published:** 2021-08-06

**Authors:** Björn Wullt, Daniel S.C. Butler, Ines Ambite, Julia Kinsolving, Christian Krintel, Catharina Svanborg

**Affiliations:** Department of Microbiology, Immunology and Glycobiology, Institute of Laboratory Medicine, Lund University, Lund, Sweden

**Keywords:** Bladder pain syndrome, Interstitial cystitis, Immunotherapy, Interleukin-1 receptor antagonist

## Abstract

**Background:**

Patients with bladder pain syndrome experience debilitating pain and extreme frequency of urination. Numerous therapeutic approaches have been tested, but as the molecular basis of disease has remained unclear, specific therapies are not available.

**Objective:**

Recently, a systematic gene deletion strategy identified interleukin-1 (IL-1) hyperactivation as a cause of severe cystitis in a murine model. Treatment with an IL-1 receptor antagonist (IL-1RA) restored health in genetically susceptible mice, linking IL-1–dependent inflammation to pain and pathology in the bladder mucosa. The study objective was to investigate whether IL-1RA treatment might be beneficial in patients with bladder pain syndrome.

**Design, setting, and participants:**

Patients diagnosed with bladder pain syndrome were invited to participate and subjected to daily IL-1RA injections for 1 wk, followed by a treatment break. Patients with other urological disorders accompanied by pain were included as controls.

**Outcome measurements and statistical analysis:**

When symptoms returned, treatment was resumed and responding patients were maintained on treatment long term, with individualized dosing regimens. Symptom scores were recorded and molecular effects were quantified by neuropeptide and gene expression analysis. DNA samples were subjected to exome genotyping.

**Results and limitations:**

IL-1RA treatment reduced bladder pain and the frequency of urination in 13/17 patients (*p* < 0.001). Substance P levels in urine were lowered, and responders returned to a more normal lifestyle. Neuroinflammatory-dependent and IL-1–dependent gene networks were inhibited, as well as regulators of innate immunity. Genotyping revealed disease-associated *IL1R1, NLRP3*, and *IL1RN* DNA sequence variants in the responders. Controls did not benefit from IL-1RA treatment, except for one patent with cystitis cystica.

**Conclusions:**

In this clinical study, IL-1RA treatment is proposed to reduce chronic bladder pain, immediately and in the long term. Despite the limited number of study patients, the potent acute effect and lasting symptom relief indicate that this therapeutic approach may be worth exploring in controlled clinical trials.

**Patient summary:**

Treatment with an interleukin-1 (IL-1) receptor antagonist is proposed for treating bladder pain syndrome, as it can result in symptom relief and increase quality of life. Reduced neuroinflammation and IL-1 signaling provided molecular evidence of the treatment effects.

## Take Home Message

Interleukin-1 (IL-1) receptor antagonist immunotherapy is proposed as a new approach to treating bladder pain syndrome, a debilitating disorder. Treated patients experienced symptom relief and increased quality of life. Reduced neuroinflammation and IL-1 signaling provided molecular evidence of the treatment effects.

## 1 Introduction

Immune response to infection must be controlled tightly to achieve protection without collateral damage. Immune balance is easily lost, however, as host deficiencies may cause the immune defense to underperform or overachieve. Immunotherapy has the potential of restoring balance to the immune system, by compensating deficiencies or inhibiting excessive immune activation and progression to chronic disease [1], [2], [3].

The bladder pain syndrome (BPS) has been associated with urinary tract infection (UTI) susceptibility [4], [5] and patients experience debilitating pain at bladder filling, extreme frequency of urination, and urgency of micturition, which destroy careers, social life, and sexual health [6], [7]. Current therapeutic options are unfortunately insufficient. Peroral analgesics including opioids are mostly not effective, and numerous experimental intravesical treatments have shown little or no long-term effect [8]. Bladder lesions may be removed endosurgically, but in many cases, surgery has no lasting effect on bladder pain. The quality of life is severely impaired in this patient group [6], [9]. The molecular basis of the BPS has remained an enigma, however, precluding the development of more specific therapies [10].

Molecular studies have identified specific innate immune checkpoints that control the severity of UTI and serve as therapeutic targets in animal models of acute pyelonephritis and acute cystitis [11], [12], [13]. Immunotherapy is therefore emerging as an alternative to antibiotics in UTI [11], [12]. Interleukin-1 (IL-1) hyperactivation has been identified as a molecular cause of acute cystitis and bladder pathology [11], also affecting the pain receptor NK1R and its ligand substance P [11], [12]. The natural IL-1 receptor antagonist (IL-1RA) inhibits the binding of IL-1 to its receptor and is used clinically as a biological immunomodulant, with an excellent safety record for indications such as gout and rheumatoid arthritis [14], [15]. In proof-of-concept studies, recombinant IL-1RA was shown to dramatically reduce bladder inflammation and the pain response in susceptible mice during acute cystitis, and to accelerate bacterial clearance [11]. This study addressed whether similar molecular mechanisms may be activated in patients with BPS and whether IL-1RA therapy might be relevant in this patient group.

## 2 Patients and methods

### 2.1 Patients

Patients diagnosed with severe BPS (O’Leary-Sant interstitial cystitis index >6) [16], [17] were prospectively recruited and offered off-label treatment with Anakinra (Kineret; Swedish Orphan Biovitrum). A diagnosis of BPS was based on the diagnostic criteria and classification defined by van de Merwe et al [18] and the European Association of Urology (EAU) [10]. All patients had persistent or recurrent pain from the bladder region, combined with worsening of symptoms during bladder filling and an abnormal frequency of micturition (median symptom duration 5.8 yr, range <1–>20 yr). The patients underwent cystoscopy at enrolment and were classified according to the presence or absence of Hunner’s lesions [10]. The diagnostic workup was retrospective, and patients had undergone hydrodistension under general anesthesia, in connection with earlier cystoscopy or endosurgery (Table 1 and Supplementary Table 1). Lesions had been confirmed by histology, detecting inflammation and/or detrusor mastocytosis compatible with BPS type 3C [19]. Exclusion criteria included ongoing UTI.

**Table 1:** Patient characteristics, summary

Characteristic	BPS type 1A	BPS type 3C	Total
	(*n* = 7)	(*n* = 10)	(*n* = 17)
Age, median (range)	66 (44–93)	66 (39–77)	66 (39–93)
Sex, *n* (%)			
Male	3 (43)	4 (40)	7 (41)
Female	4 (57)	6 (60)	10 (59)

Previous treatment, *n* (%) a			
NSAIDs	7 (100)	10 (100)	17 (100)
Nonopioid treatment	1 (14)	2 (20)	3 (17.6)
Opioid treatment	4 (57)	4 (40)	8 (47)
Intravesical instillations b	3 (43)	3 (30)	6 (35)
TURB	–	9 (90)	9 (53)

BPS = bladder pain syndrome (classification after European Guidelines [2019] and de Merwe et al [18]); NSAID = nonsteroidal anti-inflammatory drug; TURB = transurethral resection of the bladder.

See also Supplementary Table 1 for full patient characteristics.

a Patients reported insufficient pain killing effects by any pain medication previously tried except for patient PVI who had long-lasting effect of intermittent per-oral corticosteroids. All patients tried NSAID and paracetamol. Analgesics were used in some patients who choose to continue despite insufficient effect. Two patients (P1 and AA) use regularly per-oral morphine due to severe pain, but also with insufficient effect.

b Different intravesical drugs were tried, including Gepan/Hyacyst/Uracyst (chondroitin sulfate), Ialuril (hyaluronic acid + chondroitin sulfate), and dimethyl sulfoxide. Intravesical injection of Botox was not tried in any patient.

Seventeen patients were included in the study (seven males and ten females, median age 66 yr, range 39–93 yr, seven with BPS type 1A and ten with BPS type 3C). For a summary of patient characteristics, see Table 1, and for detailed individual information, see Supplementary Table 1. All 17 patients were subjected to a detailed clinical evaluation as well as symptom scoring before and repeatedly after the start of IL-1RA treatment, as well as urine neuropeptide measures. The first ten patients were further subjected to a detailed analysis of the disease response by gene expression analysis of RNA repeatedly after the start of IL-1RA treatment and genetic analysis by DNA exome sequencing.

Four patients diagnosed with other urological diseases were enrolled as controls. They were diagnosed with urethritis, prostate pain, chronic pelvic pain syndrome, or cystitis cystica (Supplementary Table 4).

### 2.2 Ethics statement

Collection of samples and analysis of data were approved by the Regional Ethics Committee, Lund, and the Swedish Ethical Review Authority (2013-004961-14 and 2019-02746), and was performed in accordance with the principles of the Declaration of Helsinki. All included patients had read and signed informed consent forms.

### 2.3 Study design

**Fig. 1:**
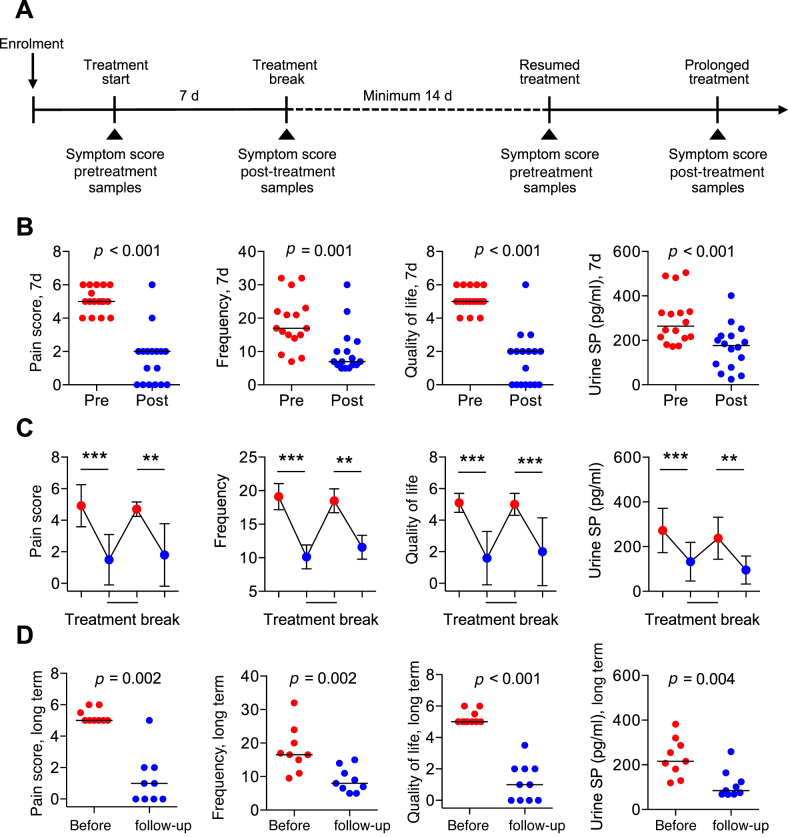
Off-label IL-1RA treatment in patients with bladder pain syndrome. (A) Overview of the treatment protocol. Patients with a history of bladder pain syndrome and ongoing disease were offered IL-1RA treatment, off-label. IL-1RA was administered once daily for 7 d. Treatment was transiently interrupted and then resumed when symptoms returned. Long-term treatment regimens were adjusted to the individual response pattern (Supplementary Table 2). Treatment effects were evaluated as changes in pain score, voiding frequency, and quality of life, using a questionnaire in Swedish and the O’Leary score (Table 2). (B) A significant reduction in pain score and voiding frequency, and an increase in the quality of life were detected after the first 7 d of treatment. Urine Substance P levels were reduced. (C) Bimodal treatment response, showing a return of symptoms during the treatment break and an increase in Substance P levels, followed by symptom relief and inhibition of Substance P release, when treatment was resumed. (D) Analysis of long-term efficacy, defined by a reduction in pain score and voiding frequency and increased quality of life. Horizontal bars indicate group medians. Data in Figures 1B and 1 D are shown for individual patients. Data in Figure 1C are shown as group means at each time point. Changes are evaluated by Wilcoxon’s signed rank test. The red color indicates pretreatment samples and blue indicates post-treatment samples. IL-1RA = interleukin-1 receptor antagonist; SP = Substance P.

Off-label Anakinra treatment was initiated after the receipt of written informed consent. The patients received daily subcutaneous Anakinra injections for 7 d (100 mg/dose). On day 7, treatment was transiently interrupted until symptoms reappeared. The patients were then invited to continue IL-1RA treatment or leave the study (Fig. 1A). The frequency of treatment was adjusted to clinical need of the patient (Table 2 and Supplementary Table 2). Side effects were recorded and compared with the known side-effect profile of the registered drug Kineret (Supplementary Table 3).

**Table 2:** Symptom scoring and summary

	Responders	Nonresponders	Total
BPS type 1A	6	1	7
BPS type 3C	7	3	10
Total, *n* (%)	13 (76.5)	4 (23.5)	17 (100)
Immediate response			
Onset (h)	4.2	–	–
Duration (d)	9.1	–	–
*Treatment effects*			
Frequency (night)			
Before (Pre)	13.6 (4.3)	13.8 (3.8)	12.9 (4.3)
After (Post)	6.1 (1.5) **, ***	13.3 (3.4) ^NS^	8.0 (1.9) **, ***
Pain			
Before (Pre)	5.2	5.1	5.1
After (Post)	1.0 ***	4.4 ^NS^	1.8 ***
Quality of life			
Before (Pre)	5.2	5.0	5.1
After (Post)	1.0 ***	4.3 ^NS^	1.8 ***
O'Leary symptom index			
Before (Pre)	17.4	17.5	17.4
After (Post)	4.6 **	16.0 ^NS^	7.2 ***
O'Leary problem index			
Before (Pre)	15.4	15.0	15.4
After (Post)	3.9 **	15.0 ^NS^	6.5 ***
Long-term treatment observation			174.6

BPS = Bladder pain syndrome; NS = nonsignificant (Wilcoxon signed rank test).

See also Supplementary Table 2 for full details.

** *p* < 0.01, Wilcoxon signed rank test.

*** *p* < 0.001, Wilcoxon signed rank test.

### 2.4 Symptom score

To document treatment effects, patients were interviewed at each visit by the study physician. Symptom scores were recorded by the patients, using a self-assessment questionnaire in Swedish. The patients registered voiding frequency during 24 h and nocturia (mean frequency during 1 wk), and a questionnaire of global scoring for local pain and quality of life. Scoring was between 0 and 6, with 0 indicating no pain/high quality of life and 6 indicating severe pain/low quality of life (Supplementary material). In addition, patients completed the O’Leary symptom and problem questionnaire in English [16]. If language support was required, patients were assisted by the clinical staff. Catheter-treated patients (*n* = 3) were assessed only by the Swedish questionnaire, as the voiding frequency parameter in the O’Leary score could not be determined.

### 2.5 Molecular analyses

Urine samples were obtained at the time of diagnosis and at each visit, during the study. Urine cultures were performed and leucocyte esterase was quantified. Urine Substance P levels were quantified using the commercially available Substance P parameter assay kit (R&D System) as per the manufacturer’s instructions (detection limit 31.5 pg/ml). Urine samples for Substance P analysis were also collected from six healthy controls.

Blood samples were obtained for DNA extraction at the onset of the study. Blood samples for RNA extraction were obtained on days 0 and 7, and repeatedly during follow-up.

For gene expression analysis, RNA was purified from peripheral blood using Tempus blood RNA tubes and purification kit (Applied Biosystems) and subjected to expression microarray analysis: 100 ng of total RNA was amplified using GeneChip 3′ IVT PLUS or GeneChip WT PLUS reagent kits. After fragmentation and labeling, cRNA was hybridized to Human Genome U219 or Human Clariom S HT arrays (all from Affymetrix), either in house using the GeneAtlas system or by Eurofins Genomics. The data were normalized using Robust Multi Average implemented in the Transcriptome Analysis Console software (TAC v.4.0.1.36; Applied Biosystems). An absolute fold change of >1.5 compared with the pretreatment sample in individual patients was considered significant. Differentially expressed genes were examined using Ingenuity Pathway Analysis software (IPA; Qiagen Bioinformatic). Heatmaps were constructed using the Gitools 2.1.1 software.

DNA was extracted from peripheral blood samples using the QIAamp DNA blood kit as per the manufacturer’s instructions (Qiagen). Exome genotyping was by Illumina Infinium Exome bead chip technology with >240 000 markers from diverse populations. Single nucleotide polymorphisms (SNPs) were annotated using the Ensemble Variant Effect Predictor. ID extraction and data conversion were carried out using R [20]. The variant coordinates used as identifiers for annotation were obtained from a strand file from https://www.well.ox.ac.uk/∼wrayner/strand/index.html#Illumina (GSAMD-24v2-0_20024620_A1-b37-strand.zip file). The dbSNP IDs or coordinates not present in the annotation file were extracted from the original IDs using standard pattern matching methods. To obtain dbSNP IDs (rs numbers), the coordinates were converted to dbSNP IDs using a local copy of the dbSNP database. Moreover, the reference alleles were included in the annotations using Samtools version 1.9 and in-house Python scripts [21].

The variant allele frequency of individual SNPs within these genes were calculated based on the formula: $freqA=\;\left(2xfreq\left(AA\right)+\;freq\;\left(AB\right)\right)÷AC$, where *A* is the variant allele and *AC* is the total allele count. The *AC* was calculated across the samples by subtracting the number of missing genotype calls from the theoretical maximum allele count, which is two times the number of samples. The allele and genotype counts for the variants were retrieved from the genotype dataset using Tabix and in-house Python scripts [22]. Significantly different allele and genotype counts were determined by the Fisher's exact test implemented in R for each variant.

Patients XI–XVII were subjected to limited genotyping by pyrosequencing (PSQ96) after polymerase chain reaction amplification of chromosomal DNA using the PyroMark Gold Q96 SNP reagent. Biotinylated primers were designed to detect polymorphisms in *MMP7* (rs11225298)*, Il1R1* (rs10199359), or *TACR1* (rs3771833).

### 2.6 Statistical analysis

The data were analyzed by Prism (GraphPad version 6.01) and were considered statistically significant at *p* < 0.05. Clinical outcome variables were assessed by the Mann-Whitney *U* test and paired data using the Wilcoxon signed rank tests. Genotype data were compared using Fisher’s exact test.

## 3 Results

### 3.1 Participants and treatment protocol

Off-label IL-1RA treatment was offered to patients diagnosed with BPS (Table 1, Table 2). After informed consent, patients received daily subcutaneous injections of Anakinra (100 mg) for 7 d. Treatment was transiently interrupted, resumed when symptoms returned, and continued with individual treatment regimens (Fig. 1A). Therapeutic outcome variables were recorded using a symptom journal, detailing the frequency of micturition, global scoring of pain intensity, and quality of life, and confirmed by O’Leary scores. Cystoscopy at enrolment revealed signs of mucosal inflammation in ten patients (BPS type 3C; Table 1). The remaining seven patients showed no detectable bladder pathology (BPS type 1A; Table 1). Four patients with other urological disease conditions were included as controls. The controls suffered from lower pelvic pain, prostatitis, urethritis, or cystitis cystica (Supplementary Table 4).

### 3.2 Treatment efficacy and safety

Treatment response rates were calculated for each patient group and the two groups combined (Fig. 1B–D, Table 2, and Supplementary Table 2). The immediate subjective response of the patients with BPS was remarkable. Significant clinical improvement was observed in 14/17 patients within the first 7 d, defined by a reduction in symptom score (pain and frequency, *p* < 0.001) and increased quality of life (*p* < 0.001; Fig. 1B). One 47-yr-old patient with very severe disease since the age of 17 yr noted a relief of symptoms within 1–2 h after the first injection and reported a return to normal life (Supplementary Table 2). In the controls with lower pelvic pain, prostatitis, or urethritis, no effect was detected and treatment was discontinued after 1 wk. The patient with cystitis cystica responded and experienced long-term symptom relief.

A lasting clinical response was detected in 13/17 patients, and 11 have remained on IL-1RA treatment for a median of 222 d (range 125–365 d). Individual treatment regimens have varied from daily (four patients) to weekly (five patients) or monthly (two patients) injections. One patient stopped successful treatment after 28 d due to worsened migraine attacks, and one discontinued treatment after 80 d due to a waning effect (Supplementary Table 2).

The clinical response during the 1st week of treatment was accompanied by a reduction in Substance P levels (13/17 patients, *p* < 0.001; Fig. 1B). Substance P and its receptor NK1R are essential for pain sensing in cystitis and inhibitors of NK1R have been shown to reduce bladder inflammation and pain in mice [12]. The effect on Substance P was biphasic with an initial reduction, followed by an increase when treatment was interrupted and a second decrease when therapy was resumed (Fig. 1C). Samples obtained during long-term follow-up (days 125–365) showed that the urine Substance P levels remained low compared to day 0 (Fig. 1D). In contrast, the control group with other urological disorders had low urine Substance P at enrollment, with little or no change after IL-1RA treatment, except for the patient with cystitis cystica (Supplementary Fig. 1).

### 3.3 Molecular validation of treatment effects

**Fig. 2:**
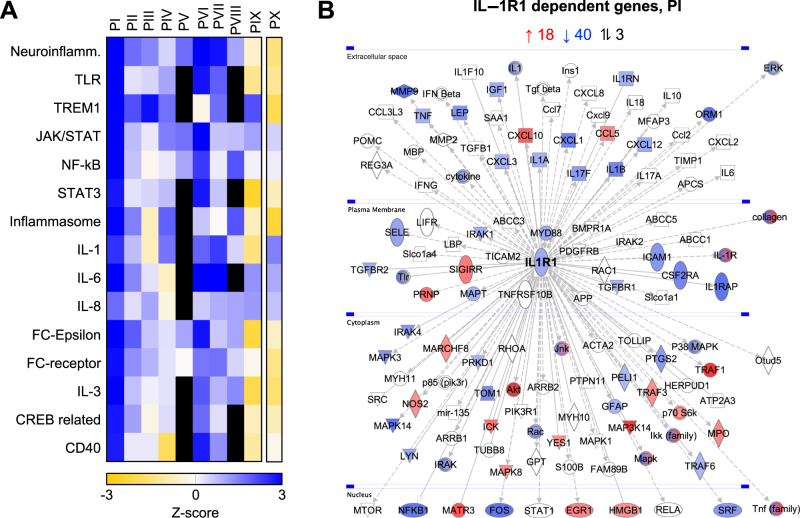
IL-1RA treatment inhibits the expression of proinflammatory genes in patients with bladder pain syndrome. (A) Gene expression analysis of RNA samples obtained before and 7 d after the onset of IL-1RA treatment. Samples from days 0 and 7 were compared intraindividually. In the heatmap of regulated pathways in individual patients, orange color indicates activated and blue inhibited (cutoff Z score >1.5). In the responders (PI-PIX), neuroinflammation, IL-1, and inflammasome signaling pathways were inhibited, as well as Toll-like receptor (TLR) recognition and adaptive immunity. (B) IL-1R1–dependent gene network, showing a significant inhibitory effect of treatment (blue indicates downregulated genes and red upregulated genes). Data are shown for patient I (for patients II–X, see Supplementary Fig. 2). See also Supplementary Fig. 4. IL = interleukin; IL-1RA = IL-1 receptor antagonist.

Genome-wide transcriptomic analysis was performed to further understand the molecular basis of the treatment response. RNA samples from the first ten patients were compared at onset, when the patients were in pain and after 1 wk of treatment (Fig. 2A). Gene expression was inhibited by treatment in eight of ten patients, including canonical pathways involved in neuroinflammation, Toll-like receptor (TLR)-dependent pattern recognition, and IL-1 signaling. IL-1R1–dependent gene expression was inhibited, including *IL1R1* itself, *IL1RAP*, *CXCL1*, and *CXCL3* (Fig. 2B and Supplementary Fig. 2). The patient who discontinued treatment after 80 d did not show this gene expression pattern. One patient with a clinical response showed a decrease in Substance P, but no effect on gene expression.

**Fig. 3:**
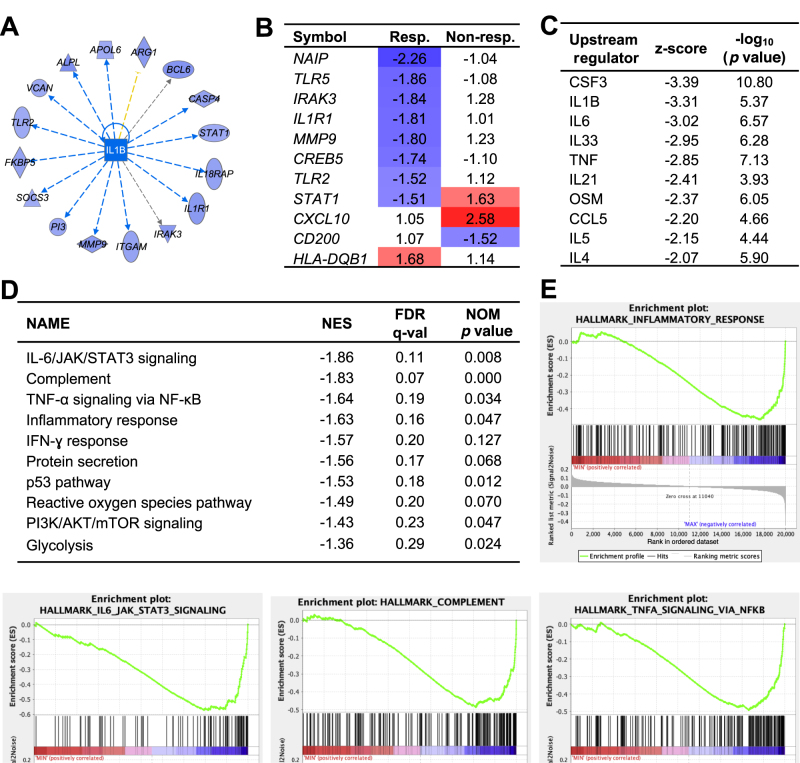
Groupwise transcriptomic response. (A) Network of IL-1β–dependent genes, which were inhibited. Il-1β–dependent genes were predicted to be inhibited in responding patients. (B) Neuroinflammatory genes inhibited by IL-1RA treatment in clinical responders. (C) Upstream regulators of the response include cytokines. (D) GSEA analysis of cellular functions modified IL-1RA treatment. A negative NES reflects an inhibition of associated functions. Significantly inhibited gene sets included IL-6/JAK/STAT3, TNF-α, and complement functions. No gene set was significantly activated. (E) Enrichment plots of the inflammatory response gene set. Enrichment plots of the four most strongly regulated gene sets are shown. Gene sets with a false discovery rate (FDR) of <25% and nominal *p* < 0.05 are considered significantly enriched. GSEA = gene set enrichment analysis; IFN = interferon; IL = interleukin; IL-1RA = IL-1 receptor antagonist; NES = normalized enrichment score, compared with presamples; NOM = nominal; Non-resp. = nonresponder; Resp. = responder; TNF = tumor necrosis factor.

Gene expression analysis of the combined data set identified neuroinflammatory genes as significantly inhibited by treatment. These included the alternative inflammasome constituent NAIP, TLRs 2 and 5, IRAK3, and the IL-1 receptor 1 (Fig. 3A and 3 B). Upstream regulator analysis identified IL-1β, IL-6, IL-33, and CSF3 as inhibited (Fig. 3C). In addition, the combined gene set enrichment analysis revealed a more general inhibitory effect on innate immunity, involving JAK/STAT, IFNs, and TNF gene sets (Fig. 3D and 3 E).

### 3.4 Genetic analysis of BPS

In the experimental UTI model, the most severe form of acute cystitis occurs in mice, lacking *Asc* or *Nlrp3*, which are inflammasome constituents [11], [23]. Excessive pro-IL-1β processing by the metalloproteinase MMP-7 drives pathology in these mice. Mice lacking IL-1β, in contrast, are protected from acute cystitis, illustrating the importance of IL-1 for symptoms and pathology [11].

Patient DNA sequence variants were identified by whole-exome genotyping. Potential susceptibility genes were selected for analysis, based on known disease associations in the murine cystitis model (*IL1A*, *IL1B*, *IL1RN*, *IL1R1*, *NLRP3*, *PYCARD*, *MMP7*, *TAC1*, and *TACR1*) [11], [12].

**Fig. 4:**
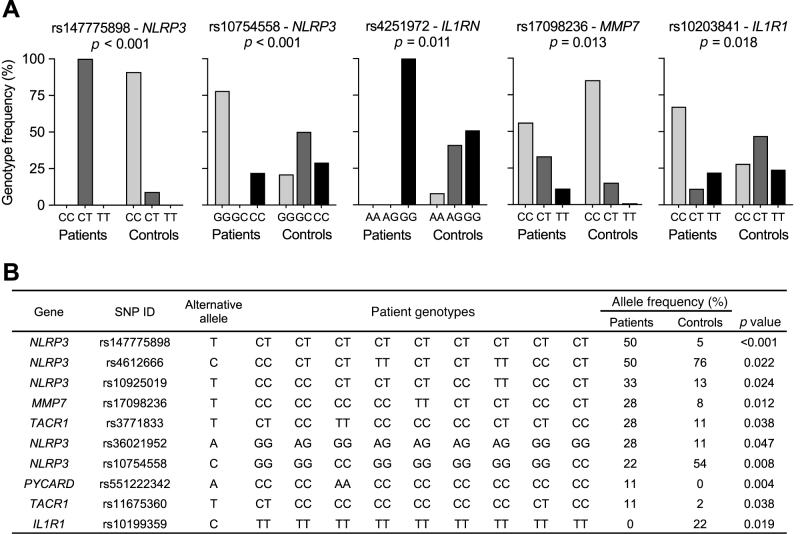
Genetic analysis of patients with bladder pain syndrome. (A) Exome genotyping identified disease-associated SNPs in the responders, compared with the 1000 genome control database (European population). Disease-associated SNPs were found in in *NLRP3* (rs147775898 and rs10754558), *IL1RN* (rs4251972), *MMP7* (rs17098236), and *IL1R1* (rs10203841). (B) Table of allele frequencies of disease-associated SNPs, ranked by *p* value. Data were analyzed using the Fisher’s exact test. SNP = single nucleotide polymorphism.

Disease-associated genetic variants were identified by comparing with the 1000 genome data for the European population [21] (Fig. 4 and Supplementary Fig. 3). Fourteen significant SNPs were detected within *IL1R1*, *NLRP3*, *PYCARD*, *MMP7*, and *TACR1*. *PYCARD* encodes the inflammasome constituent ASC, identified as a cystitis susceptibility gene in mice [11]. *TACR1* encodes the Substance P receptor NK1R, which drives pain sensing from the urinary bladder to dorsal root ganglia [12]. The *MMP7* variant rs17098236 has been associated with gout and HIV infection but not functionally characterized [24], [25]. The detected variants were intronic, suggesting potential regulatory rather than structural effects. For analyses of gene expression and DNA sequence variants in the last seven patients, please see Supplementary Figure 4.

For side effect analysis, please see Supplementary Table 3. Acute local skin reactions were reported in five out of 17 patients. During long-term treatment, mild to moderate neutropenia or transient liver enzyme reactions were each observed in one patient. After a treatment break and normalization of the parameters, IL-1RA treatment was resumed, according to standard practice [26]. Headache after injection was reported by one patient.

## 4 Discussion

Innate immunity controls the response to infection at mucosal surfaces, where the initial contact between microbes and hosts usually takes place [27]. The damaging potential of an imbalanced response is highly significant, not least in the urinary tract, where excessive immune activation has been identified as a cause of pathology in acute pyelonephritis and acute cystitis. Immunotherapy has therefore been tested, and found to return the balance and defensive efficiency of the immune response. Small RNA-based inhibition of the IRF-7 transcription factor was effective in a murine pyelonephritis model [13], and IL-1RA therapy prevented severe cystitis and accelerated bacterial clearance in an acute cystitis model [11]. These findings provided the rationale to initiate off-label IL-1RA therapy in patients with BPS. Significant clinical improvement was detected in 76% of the patients after 1 wk and the beneficial effects were found to continue with long-term treatment, in several patients. Placebo controlled trials might therefore be undertaken to fully evaluate the therapeutic potential of this approach.

Placebo effects are prevalent in patients with BPS. Decreased symptom scores have been reported in up to 50% of patients receiving placebo in controlled clinical trials [19], [28]. The clinical protocol was designed with this effect in mind. Using a stop and go approach, the patients were treated for 1 wk, taken off treatment until symptoms recurred, and then offered to continue treatment. In the responders, symptoms returned when treatment was interrupted, followed by long-term symptom relief when treatment was reintroduced in 13 patients. The long-term symptom relief further supports a treatment effect, as the placebo effect is known to decrease with time [29].

## 5 Conclusions

Data from the murine UTI model suggest that when IL-1 homeostasis is restored, a vicious cycle of neuroinflammation and pain is interrupted [12]. Molecular effects of IL-1RA treatment further indicate that this may be the case also in patients with bladder pain. The findings point to BPS as a group of conditions where immune imbalances converge on IL-1 and neuropeptide hyperactivation. A genetic element of disease susceptibility was observed, potentially affecting the balance of the IL-1 response, but this needs further study. The findings suggest that IL-1R inhibition may constitute a novel, more specific treatment approach in patients with bladder pain, with the potential to improve the quality of life. Innate immunotherapy using IL-1RA may also be relevant in recurrent cystitis, where therapeutic options are becoming increasingly limited due to antibiotic resistance.

  ***Author contributions*:** Catharina Svanborg had full access to all the data in the study and takes responsibility for the integrity of the data and the accuracy of the data analysis.

  *Study concept and design*: Wullt, Butler, Ambite, Svanborg.

*Acquisition of data*: Wullt, Butler, Ambite, Kinsolving, Krintel.

*Analysis and interpretation of data*: Wullt, Butler, Svanborg.

*Drafting of the manuscript*: Wullt, Butler, Ambite, Svanborg.

*Critical revision of the manuscript for important intellectual content*: Kinsolving, Krintel.

*Statistical analysis*: Butler.

*Obtaining funding*: Butler, Svanborg.

*Administrative, technical, or material support*: Wullt, Ambite.

*Supervision*: Krintel, Svanborg.

*Other*: None.

  ***Financial disclosures:*** Catharina Svanborg certifies that all conflicts of interest, including specific financial interests and relationships and affiliations relevant to the subject matter or materials discussed in the manuscript (eg, employment/affiliation, grants or funding, consultancies, honoraria, stock ownership or options, expert testimony, royalties, or patents filed, received, or pending), are the following: The authors hold shares in SelectImmune Pharma, a biotech company, with an interest in exploring the potential of IL-1RA treatment in cystitis and bladder pain.

  ***Funding/Support and role of the sponsor*:** Support was from national grants. Financial support was generously provided by the Swedish Medical Research council (#2016-01952), the 10.13039/501100002794Swedish Cancer Society (#140776), the Österlund Foundation, the Inga-Britt and Arne Lundberg Foundation, the Hedda and John Forssman Foundation for medical research (#40964), and the Royal Physiographic Society of Lund (#136260). The European Union’s Horizon 2020 research and innovation programme (#954360) provided support to the laboratory infrastructure. Patents have been filed for the use of IL-1RA as a treatment for cystitis and bladder pain.

  ***Acknowledgments*:** We gratefully acknowledge technical support of the Genome Institute of Singapore (DNA sequencing) and Jane Pullman at Genevia Technologies Oy (annotation and exome genotyping data analysis), and clinical advice from Dr. M.C. Kapetanovic, the Rheumatology Division, Lund University Hospital, Sweden.
