# Supplementary material for: Immunomodulation—A Molecular Solution to Treating Patients with Severe Bladder Pain Syndrome?
Source: Eur Urol Open Sci. 2021 Aug 6;31:49–58. doi: 10.1016/j.euros.2021.07.003 (PMC8385293; doi:10.1016/j.euros.2021.07.003)
Supplement: Supplementary file 1 [file mmc1.docx]

**Supplementary Table 1. Patient characteristics**

|  | | | | | | | | | |  |  |  |
| --- | --- | --- | --- | --- | --- | --- | --- | --- | --- | --- | --- | --- |
| ID | Age  (years) | M/F | BPS Type ^a^ | Symptom duration (year/s) | Inflammation/  Hunner’s Ulcers | | Previous Pain  medication | Effect of previous TURB ^b^ | Concomitant diseases and treatments |  |  |  |
|  |  |  |  |  | Cystoscopy | Histology |  |  |  |  |  |  |
|  | | | | | | | | | |  |  |  |
|  | | | | | | | | | |  |  |  |
| *Responders* | | | | | | | | | |  |  |  |
|  | | | | | | | | | |  |  |  |
| PI ^c^ | 76 | F | 3C | 2 | Positive | Positive | Peroral Morphine and Cortisone | Negative | Chronic obstructive pulmonary disease  Small contracted bladder  Repeated TURB without improvement |  |  |  |
|  |  |  |  |  |  |  |  |  |  |  |  |  |
| PII | 66 | M | 3C | > 10 | Positive | Positive | NSAID, Codeine, Paracetamol, Doxycycline | Transient ^e^ | Gout, allopurinol treatment  Post endosurgery for benign prostate hyperplasia.  TURB with transient effects (2 years) |  |  |  |
|  |  |  |  |  |  |  |  |  |  |  |  |  |
| PIII ^d^ | 61 | M | 3C | 2 | Positive | Positive | Paracetamol | Negative | Partial paraparesis, neurogenic bladder  disorder, kidney stone  Repeated TURB without improvement |  |  |  |
|  |  |  |  |  |  |  |  |  |  |  |  |  |
| PIV | 66 | F | 3C | 5 | Positive | Positive | NSAID  Tramadol | Transient | Otherwise healthy  TURB with transient effects (1 year) |  |  |  |
|  |  |  |  |  |  |  |  |  |  |  |  |  |
| PV | 66 | M | 3A/C | > 2 | Positive | Positive ^f^ | NSAID  Paracetamol | Positive ^e, f^ | Gout , allopurinol treatment  Post-surgery for benign prostate hyperplasia |  |  |  |
|  |  |  |  |  |  |  |  |  |  |  |  |  |
| PVI | 61 | F | 3C | > 10 | Positive | Positive | Insufficient effect of any pain medication | Transient | Otherwise healthy,  TURB with transient effect (2 years) |  |  |  |
|  |  |  |  |  |  |  |  |  |  |  |  |  |
| PVII ^c^ | 67 | M | 3C | 5 | Positive | Positive | Morphine  Gabapentin | Negative | Surgery due to spinal stenosis, chronic back pain,  TURB without improvement |  |  |  |
|  |  |  |  |  |  |  |  |  |  |  |  |  |
| PVIII | 47 | F | 1A | > 40 | Negative | ND | NSAID  Paracetamol | ND | Otherwise healthy |  |  |  |
|  |  |  |  |  |  |  |  |  |  |  |  |  |
| PIX | 39 | F | 1A | < 1 | Negative | ND | Insufficient effect of any pain medication | ND | Migraine |  |  |  |
|  |  |  |  |  |  |  |  |  |  |  |  |  |
| PX | 70 | M | 1A | < 1 | Negative | ND | NSAID  Tramadol | ND | Prostate pain, atrial fibrillation |  |  |  |
|  |  |  |  |  |  |  |  |  |  |  |  |  |
| PXI | 70 | F | 1A | > 10 | Negative | ND | NSAID  Tramadol, Codeine | ND | Mb Dercum, overactive bladder, spinal stenosis  stenosis |  |  |  |
|  |  |  |  |  |  |  |  |  |  |  |  |  |
| PXII | 65 | M | 1A | 10 | Negative | ND | Paracetamol | -- | Overactive bladder, urge incontinence,  recurrent UTI, Betigma |  |  |  |
|  |  |  |  |  |  |  |  |  |  |  |  |  |
| PXIII | 57 | M | 1C | > 20 | Negative | Negative | NSAID | -- ^e^ | Hypertonia, chronic obstructive pulmonary disease |  |  |  |
|  |  |  |  |  |  |  |  |  |  |  |  |  |
| *Non* *responders* | | | | |  |  |  |  |  |  |  |  |
|  |  |  |  |  |  |  |  |  |  |  |  |  |
| PXIV | 77 | F | 3C | > 5 | Positive | Positive | Tramadol, Codeine  Corticosteroids | Transient ^e^ | Hypothyroidosis  Levothyroxine  TURB with transient effects (5 years) |  |  |  |
|  |  |  |  |  |  |  |  |  |  |  |  |  |
| PXV | 93 | F | 3B | 15 | Positive | Positive | Paracetamol | Transient | Disc prolapse, hypertonia. 15 years prior to enrolment  TURB with long term effect (14 years). |  |  |  |
|  |  |  |  |  |  |  |  |  |  |  |  |  |
| PXVI | 69 | F | 3C | 6 | Positive | Positive | Insufficient effect of any pain medication | Negative ^e^ | Disc prolapse, chronic neck and back pain  TURB without improvement |  |  |  |
|  |  |  |  |  |  |  |  |  |  |  |  |  |
| PXVII | 77 | F | 1A | 9 | Negative | ND | Morphin |  | Otherwise healthy. |  |  |  |
|  |  |  |  |  |  |  |  |  |  |  |  |  |
| a All patients were diagnosed according to the European Association of Urology guidelines with either BPS type 3C or BPS type 1A. All patients had symptomatic recurrences at enrolment. b Patients (PI-PIV and PX) were previously subjected to TURB including hydrodistension. At the time of inclusion in the study these patients had symptomatic recurrences, including inflammatory lesions by cystoscopy c Catheter d Clean Intermittent Catheterization (CIC)  e Previous Intra-vesical treatments with DMSO (PII), Gepan (PV) or Uracysyst/lauril (PVI) with transient or insufficient effect. f Patient underwent TURB with positive effect after 125 days and stopped IL-1RA treatment.  ID = Patient ID, BPS = Bladder pain syndrome, TURB = Transutheral resection of the bladder M = Male, F = Female, ND = No available data. | | | | | | | | | |  |  |  |

**Supplementary Table 2. Symptom scoring and long-term treatment**

|  | Immediate response | |  | Treatment effects | | | | | | | | | | | | | | | | | | | | | | | | | | | |  |  | | | | |  | | | | |  |
| --- | --- | --- | --- | --- | --- | --- | --- | --- | --- | --- | --- | --- | --- | --- | --- | --- | --- | --- | --- | --- | --- | --- | --- | --- | --- | --- | --- | --- | --- | --- | --- | --- | --- | --- | --- | --- | --- | --- | --- | --- | --- | --- | --- |
| ID | Onset  (hours) | Duration  (days) |  | Frequency 24h (night) | | | |  | | Pain | | | |  | | QoL | | | | |  | | O'Leary SI | | | |  | | O'Leary PI | | | | | |  | | Treatment  frequency | | | Long-term treatment  (Days) | | | |
|  |  |  |  | Pre | | Post | |  | | Pre | | Post | |  | | Pre | Post | | | |  | | Pre | | Post | |  | | Pre | | Post | | | |  | |  |  |  |  |  |  |  |
| *Responders* | | | | | | | | | | | | | | | | | | | | | | | | | | | | | | | | | | | | | | | | |  |  |  |
| PI ^a^ | 1-2 | 0.3-0.6 |  | Cath | | Cath | |  | | 5.5 | | 1 | |  | | 5.5 | | | 1 | |  | | Cath | | Cath | |  | | Cath | | Cath | | | |  | | Daily | | | 365 | | | |
|  |  |  |  |  | |  | |  | |  | |  | |  | |  | | |  | |  | |  | |  | |  | |  | |  | | | |  | |  | | |  | | | |
| PII | 4-6 | 1-2 |  | 12 (5) | | 5 (1) | |  | | 5 | | 2 | |  | | 5 | | | 2 | |  | | 18 | | 5 | |  | | 16 | | 4 | | | |  | | Monthly,  on demand | | | 365 | | | |
|  |  |  |  |  | |  | |  | |  | |  | |  | |  | | |  | |  | |  | |  | |  | |  | |  | | | |  | |  | | |  | | | |
| PIII ^b^ | 2 | 2-3 |  | CIC | | CIC | |  | | 5 | | 0 | |  | | 5 | | | 0 | |  | | CIC | | CIC | |  | | CIC | | CIC | | | |  | | Monthly,  on demand, | | | 184 | | | |
|  |  |  |  |  | |  | |  | |  | |  | |  | |  | | |  | |  | |  | |  | |  | |  | |  | | | |  | |  | | |  | | | |
| PIV | 1-2 (12) | 1-7 |  | 11 (3) | | 4 (1) | |  | | 6 | | 0 | |  | | 6 | | | 0 | |  | | 18 | | 3 | |  | | 16 | | 4 | | | |  | | Weekly | | | 365 | | | |
|  |  |  |  |  | |  | |  | |  | |  | |  | |  | | |  | |  | |  | |  | |  | |  | |  | | | |  | |  | | |  | | | |
| PV | 4-6 | 1-2 |  | 16 (5.5) | | 10 (4) | |  | | 5 | | 2 | |  | | 5 | | | 3.5 | |  | | 19 | | 11 | |  | | 16 | | 9 | | | |  | | Daily | | | 125 | | | |
|  |  |  |  |  | |  | |  | |  | |  | |  | |  | | |  | |  | |  | |  | |  | |  | |  | | | |  | |  | | |  | | | |
| PXI | 2 | 3 |  | 24 (8) | | 8 (2) | |  | | 6 | | 1 | |  | | 6 | | | 0 | |  | | 18 | | 4 | |  | | 16 | | 0 | | | |  | | Weekly | | | 287 | | | |
|  |  |  |  |  | |  | |  | |  | |  | |  | |  | | |  | |  | |  | |  | |  | |  | |  | | | |  | |  | | |  | | | |
| PXII ^a^ | 2-12 | 30 |  | 12 (5) | | 5 (1) | |  | | 5 | | 2 | |  | | 5 | | | 2 | |  | | Cath | | Cath | |  | | Cath | | Cath | | | |  | | Monthly,  on demand | | | 70 | | | |
|  |  |  |  |  | |  | |  | |  | |  | |  | |  | | |  | |  | |  | |  | |  | |  | |  | | | |  | |  | | |  | | | |
| PVI | 2 | 1 |  | 16 (5.5) | | 4 (1) | |  | | 5.5 | | 0 | |  | | 5 | | | 0 | |  | | 24 | | 0 | |  | | 16 | | 0 | | | |  | | Daily | | | 339 | | | |
|  |  |  |  |  | |  | |  | |  | |  | |  | |  | | |  | |  | |  | |  | |  | |  | |  | | | |  | |  | | |  | | | |
| PVII ^c^ | 1 | 8 |  | 12 (3) | | 6 (1) | |  | | 4 | | 0 | |  | | 4 | | | 0 | |  | | 11 | | 3 | |  | | 12 | | 2 | | | |  | | Weekly | | | 28 | | | |
|  |  |  |  |  | |  | |  | |  | |  | |  | |  | | |  | |  | |  | |  | |  | |  | |  | | | |  | |  | | |  | | | |
| PVIII | 2 | 2 |  | 8 (3) | | 8 (2) | |  | | 4 | | 2 | |  | | 6 | | | 2 | |  | | 19 | | 6 | |  | | 15 | | 6 | | | |  | | Monthly,  on demand | | | 365 | | | |
|  |  |  |  |  | |  | |  | |  | |  | |  | |  | | |  | |  | |  | |  | |  | |  | |  | | | |  | |  | | |  | | | |
| PIX | 2 | 3 |  | 12 (5) | | 6 (1) | |  | | 4 | | 0 | |  | | 4 | | | 0 | |  | | 19 | | 9 | |  | | 16 | | 9 | | | |  | | Daily | | | 365 | | | |
|  |  |  |  |  | |  | |  | |  | |  | |  | |  | | |  | |  | |  | |  | |  | |  | |  | | | |  | |  | | |  | | | |
| PXIII | 12 | 7 |  | 12 (3) | | 5 (1) | |  | | 6 | | 1.0 | |  | | 6 | | | 1 | |  | | 15 | | 3 | |  | | 16 | | 2 | | | |  | | Monthly,  on demand | | | 128 | | | |
|  |  |  |  |  | |  | |  | |  | |  | |  | |  | | |  | |  | |  | |  | |  | |  | |  | | | |  | |  | | |  | | | |
| PXIV | 4 | 14 |  | 15 (3) | | 6 (1) | |  | | 5 | | 2 | |  | | 5 | | | 2 | |  | | 13 | | 2 | |  | | 15 | | 3 | | | |  | | Weekly | | | 42 | | | |
| *Non-responders* | | | | | | | | | | | | | | | | | | | | | | | | | | | | | | | | | | | | | | | | |  |  |  |
| PX | 2-4 | 3 |  | 8 (3) | 6 (1) | |  | | 5 | | 2 | |  | | 5 | | | 2 | |  | | 18 | | ND | |  | | 13 | | ND | | | |  | | Weekly | | | 80 | | |  |  |
|  |  |  |  |  |  | |  | |  | |  | |  | |  | | |  | |  | |  | |  | |  | |  | |  | | | |  | |  | | |  | | |  |  |
| PXV | ND | ND |  | 5 (2) | 5 (2) | |  | | 4 | | 4 | |  | | 4 | | | 4 | |  | | 16 | | 16 | |  | | 13 | | 13 | | | |  | | -- | | | -- | | |  |  |
|  |  |  |  |  |  | |  | |  | |  | |  | |  | | |  | |  | |  | |  | |  | |  | |  | | | |  | |  | | |  | | |  |  |
| PXVI | ND | ND |  | 18 (4) | 18 (4) | |  | | 5.5 | | 5.5 | |  | | 5 | | | 5 | |  | | 18 | | 16 | |  | | 18 | | 16 | | | |  | | -- | | | -- | | |  |  |
|  |  |  |  |  |  | |  | |  | |  | |  | |  | | |  | |  | |  | |  | |  | |  | |  | | | |  | |  | | |  | | |  |  |
| PXVII | ND | ND |  | 24 (6) | 24 (6) | |  | | 6 | | 6 | |  | | 6 | | | 6 | |  | | 18 | | 16 | |  | | 18 | | 16 | | | |  | | -- | | | -- | | |  |  |
|  |  |  |  |  |  | |  | |  | |  | |  | |  | | |  | |  | |  | |  | |  | |  | |  | | | |  | |  | | |  | | |  |  |
| *Mean* |  |  |  | 12.9 (4.3) | 8.0 (1.9) | |  | | 5.1 | | 1.8 | |  | | 5.1 | | | 1.8 | |  | | 17.4 | | 7.2 | |  | | 15.4 | | 6.5 | | | |  | |  | | | 222 | | |  |  |
|  | | | | | | | | | | | | | | | | | | | | | | | | | | | | | | | | | | | | | | | | |  |  |  |
| a Indwelling catheter (Cath), O'Leary score not possible b Clean Intermittent catheterization (CIC), O'Leary score not possible c Patient had a complete response on IL-1RA treatment but stopped treatment due to worsened migraine attacks as side effect. d Patient stopped responding to IL-1RA treatment on day 80  A clinical response was defined based on (1) a normalized O’Leary score (<6) or (2) normalized global score (Pain and QoL = 0-1) or (3) stopped pain medication. Non-responders were defined by no persistent reduction of either O’Leary scores, or global scores. Frequency, Pain and QoL was scored based on a global questionnaire (Supplementary Info 1) and validated by O’Leary scores  ID = Patient ID, QoL = Quality of Life, ND= No data, SI = Symptom Index, PI = Problem Index | | | | | | | | | | | | | | | | | | | | | | | | | | | | | | | | | | | | | | | | |  |  |  |

**Supplementary Table 3. Reported side effects**

| **ID** |  | **Local skin irritation** | **Other** |
| --- | --- | --- | --- |
| PI |  | Not reported | Not reported |
| PII |  | Yes, initially | Transient neutropenia ^a^ |
| PIII |  | Not reported | Not reported |
| PIV |  | Yes, initially | Not reported |
| PV |  | Yes, initially | Not reported |
| PVI |  | Not reported | Transient neutropenia ^b^ |
| PVII |  | Not reported | Headache post injection, Transient liver enzyme reaction |
| PVIII |  | Not reported | Not reported |
| PIX |  | Yes, initially | Transient liver enzyme reaction |
| PX |  | Not reported | Not reported |
| PXI |  | Not reported | Not reported |
| PXII |  | Not reported | Not reported |
| PXIII |  | Yes | Transient liver enzyme reaction |
| PXIV |  | Not reported | Not reported |
| PXV |  | Not reported | Not reported |
| PXVI |  | Not reported | Not reported |
| PXVII |  | Not reported | Not reported |
| Levels below 0.5 are considered clinically relevant.  ^a^ White blood count of 1.3  ^b^ White blood count of 0.9 | | | |

**Supplementary Table 4. Patient characteristics, control group**

|  |  |  |  |  |  |  |  |
| --- | --- | --- | --- | --- | --- | --- | --- |
|  |  |  |  | Symptom | Inflammation/ | |  |
|  | Age  (years) |  | Diagnosis ^a^ | duration | Hunner’s Ulcers | | Concomitant diseases and treatments |
| ID |  | M/F |  | (year/s) | Cystoscopy | Histology |  |
|  |  |  |  |  |  |  |  |
| CI | 21 | F | Urethral Pain Syndrome | >1 | Negative | ND | Otherwise healthy |
|  |  |  |  |  |  |  |  |
| CII | 70 | M | Prostate Pain Syndrome | >10 | Negative | ND | Arthralgia UNS, arthrosis  Anti-inflammatory drugs |
|  |  |  |  |  |  |  |  |
| CIII | 55 | M | Pelvic Pain Syndrome | >10 | Negative | ND | Chronic fatigue syndrome  Intermittent antidepressant treatment |
|  |  |  |  |  |  |  |  |
| CIV ^b^ | 65 | M | Cystitis Cystica | >10 | Positive | Cystitis cystica | Unspecified intermittent fever, Arthralgia |
|  |  |  |  |  |  |  |  |
| ^a^ All pain syndromes were diagnosed using the criteria defined by the EAU-guidelines.  ^b^ Patient C4 was diagnosed by typical findings at cystoscopy, confirmed by benign histology with signs of mucosal inflammation. | | | | | | | |
