# Supplementary material for: Immunomodulation—A Molecular Solution to Treating Patients with Severe Bladder Pain Syndrome?
Source: Eur Urol Open Sci. 2021 Aug 6;31:49–58. doi: 10.1016/j.euros.2021.07.003 (PMC8385293; doi:10.1016/j.euros.2021.07.003)
Supplement: Supplementary file 2 [file mmc2.pdf]

Anakinra – Kineret behandling av cystit - pilotstudie

……………………………………… *Datum* …………………………
Namn

……………………………………..
Personnummer

Får du just nu behandling med Anakinra? ………..ja/ nej………

Om du blir behandlad:

Datum och ungefärligt klockslag för senaste injektion av Anakinra? ....

Hur många injektioner behöver du per vecka för att bli så bra som möjligt? ....inj/v

Hur snabbt märker du behandlingseffekt efter en injektion: ……………dagar

Symtom-score

1. Frekvens- ungefär hur många gånger måste Du tömma blåsan – försök ange ungefärligt medelvärde för två dagar:

Hela dygnet (inklusive natt)………………… endast på natten:…………………..

2. Smärta - lokalt från blåsan, även i samband med blåstömning – ringa in den siffra som stämmer bäst:

0 – helt besvärsfri
1 – mycket måttliga besvär
2 – måttliga besvär
3 – måttligt ont
4 – ont
5 – mycket ont
6 - outhärdligt ont

3. Om du hade haft kvar dina besvär som du just beskrivit ovan - hur hade du då beskrivit din situation?- ringa in den siffra som stämmer bäst:

0 – mycket nöjd
1 – nöjd
2 – ganska nöjd
3 – blandade känslor
4 – ganska missbelåten
5 – olycklig
6 - fruktansvärt olycklig
