# Supplementary material for: Immunomodulation—A Molecular Solution to Treating Patients with Severe Bladder Pain Syndrome?
Source: Eur Urol Open Sci. 2021 Aug 6;31:49–58. doi: 10.1016/j.euros.2021.07.003 (PMC8385293; doi:10.1016/j.euros.2021.07.003)
Supplement: Supplementary file 3 [file mmc3.pdf]

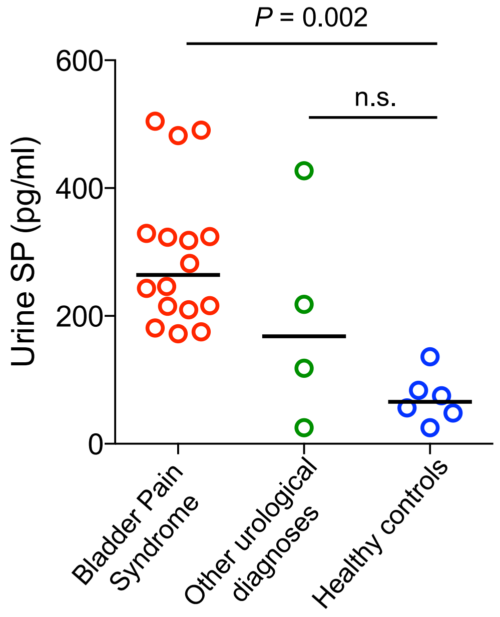

Supplementary Figure 1. Urine Substance P levels prior to treatment in patients and study controls.

Urine Substance P levels were measured by ELISA in patients with bladder pain syndrome (n = 16) other urological diagnoses (n = 4) and compared to healthy controls without urological morbidity (n = 6). The data was analyzed by Kruskal-Wallis with Dunn's correction. n.s. = not significant. The control patient with cystitis cystica had elevated SP levels and responded to IL-1RA treatment.

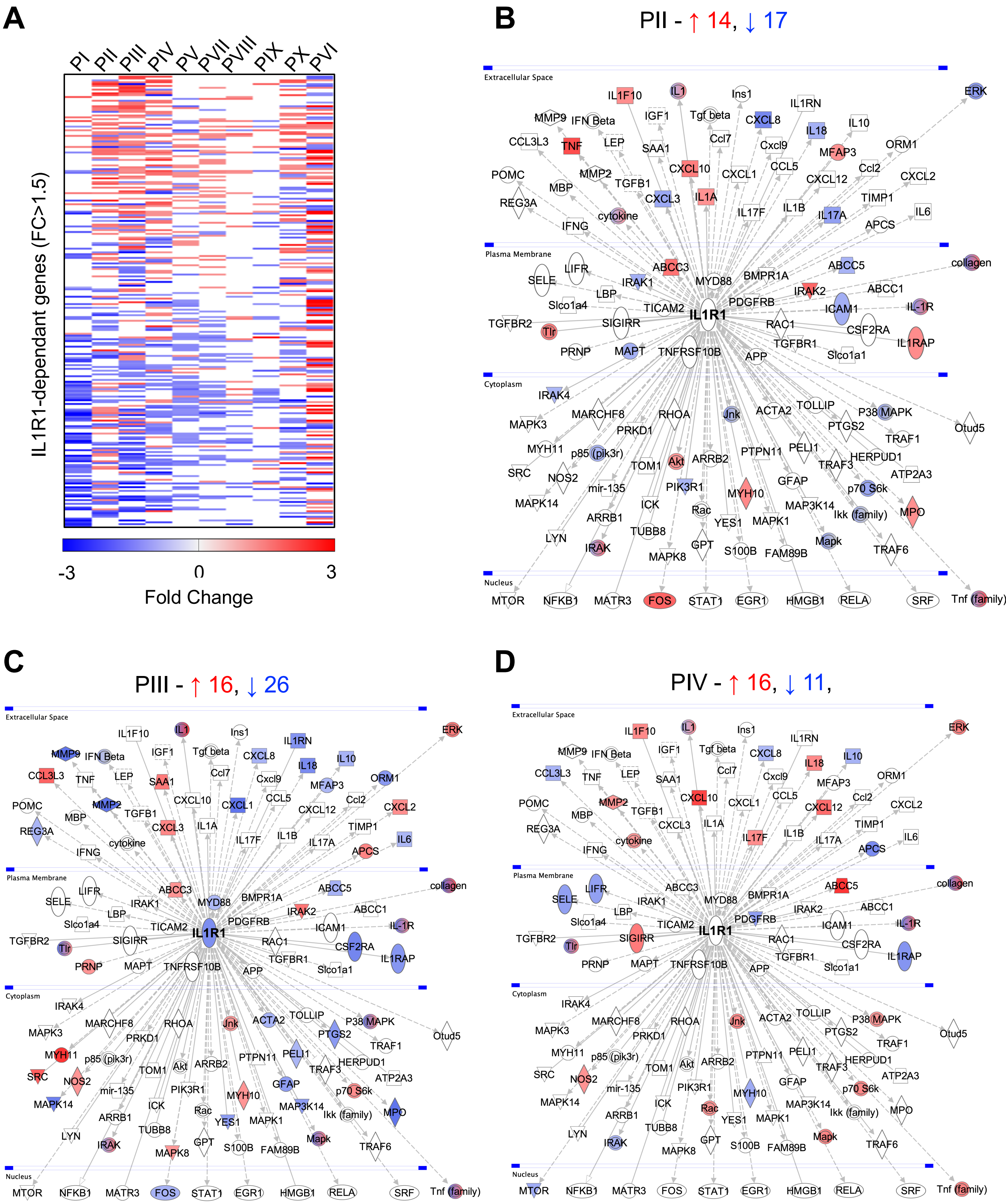
A
PI PII PIII PIV PV PVII PVIII PIX PX PVI
IL1R1-dependant genes (FC>1.5)
-3 0 3
Fold Change
B
PII - ↑ 14, ↓ 17
Extracellular Space
IL1F10 IL1 Ins1 ERK IL1RN IGF1 Tgf beta CXCL8 MMP9 IFN Beta IL18 IL10 CCL3L3 TNF LEP Ccl7 Cxcl9 ORM1 SAA1 MFAP3 CCL5 Ccl2 POMC MMP2 CXCL10 CXCL1 CXCL12 CXCL2 MBP TGFB1 CXCL3 IL1A TIMP1 REG3A IL1B IL6 cytokine IL17F IL17A APCS IFNG
Plasma Membrane
collagen ABCC5 LIFR ABCC3 SELE IRAK1 MYD88 BMPR1A IRAK2 ABCC1 LBP PDGFRB IL-1R Slco1a4 TICAM2 ICAM1 IL1R1 TGFBR2 SIGIRR RAC1 CSF2RA Tlr IL1RAP TGFBR1 PRNP MAPT TNFRSF10B APP Slco1a1
Cytoplasm
IRAK4 TOLLIP MARCHF8 RHOA Jnk ACTA2 P38 MAPK Otud5 MAPK3 PRKD1 PTGS2 TRAF1 MYH11 p85 (pik3r) PELI1 HERPUD1 Akt ARRB2 PTPN11 TRAF3 SRC TOM1 NOS2 GFAP ATP2A3 mir-135 PIK3R1 p70 S6k MAPK14 MYH10 ICK MAP3K14 Ikk (family) MPO Rac ARRB1 YES1 MAPK1 TUBB8 Mapk LYN GPT TRAF6 IRAK S100B FAM89B MAPK8
Nucleus
MTOR NFKB1 MATR3 FOS STAT1 EGR1 HMGB1 RELA SRF Tnf (family)
C
PIII - ↑ 16, ↓ 26
Extracellular Space
Plasma Membrane
Cytoplasm
Nucleus
D
PIV - ↑ 16, ↓ 11,
Extracellular Space
Plasma Membrane
Cytoplasm
Nucleus

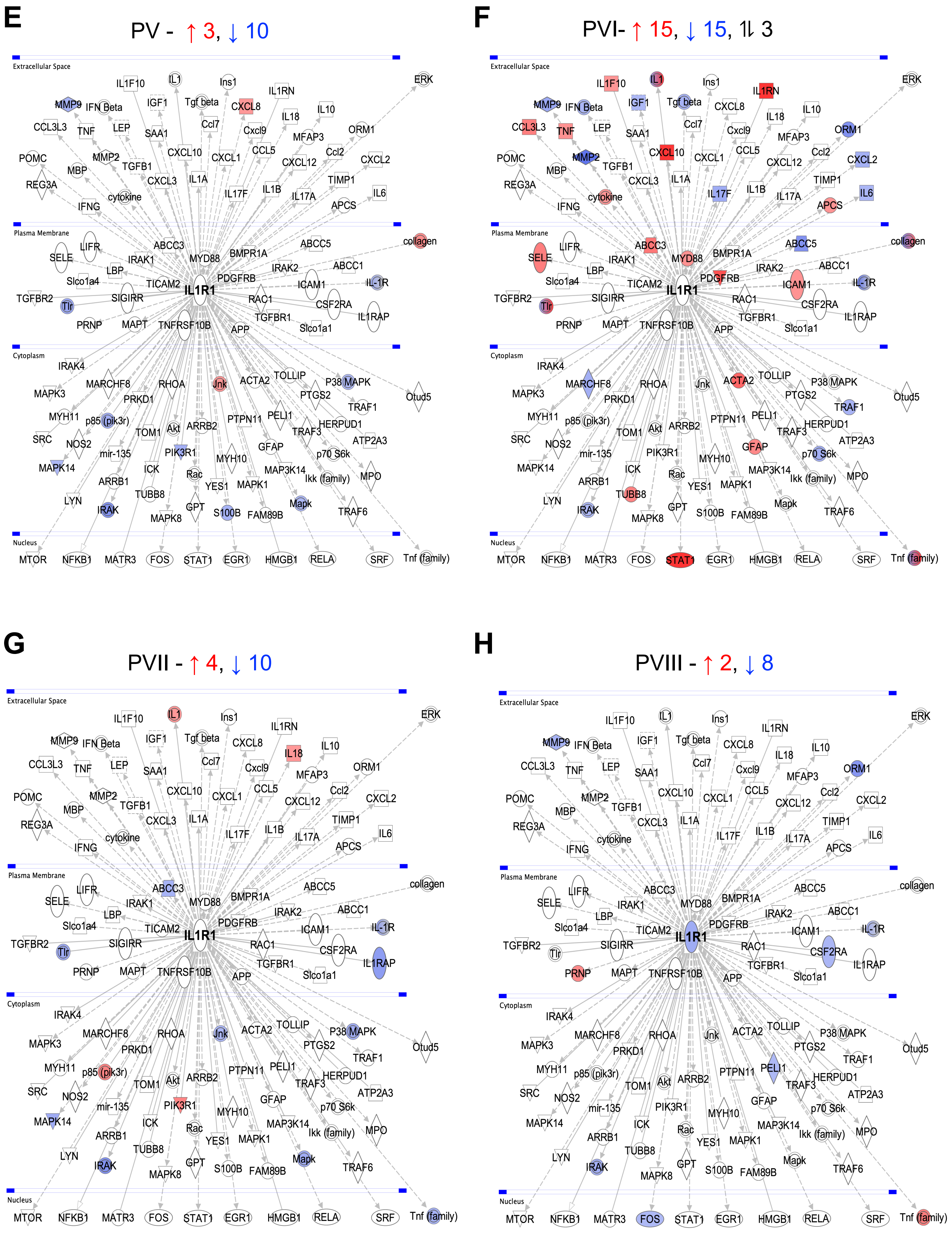
E
PV - ↑ 3, ↓ 10
F
PVI- ↑ 15, ↓ 15, ↑↓ 3
G
PVII - ↑ 4, ↓ 10
H
PVIII - ↑ 2, ↓ 8
Extracellular Space
Plasma Membrane
Cytoplasm
Nucleus
IL1R1
IL1F10
IL1
Ins1
IL1RN
ERK
MMP9
IFN Beta
IGF1
Tgf beta
CXCL8
IL18
IL10
CCL3L3
TNF
LEP
SAA1
Ccl7
Cxcl9
MFAP3
ORM1
POMC
MMP2
TGFB1
CXCL10
CXCL1
CCL5
CXCL12
Ccl2
CXCL2
MBP
CXCL3
IL1A
TIMP1
REG3A
cytokine
IL17F
IL1B
IL17A
IL6
IFNG
APCS
LIFR
ABCC3
ABCC5
collagen
SELE
IRAK1
MYD88
BMPR1A
LBP
IRAK2
ABCC1
Slco1a4
TICAM2
PDGFRB
ICAM1
IL-1R
TGFBR2
SIGIRR
RAC1
CSF2RA
Tlr
TGFBR1
IL1RAP
PRNP
MAPT
TNFRSF10B
APP
Slco1a1
IRAK4
MARCHF8
RHOA
Jnk
ACTA2
TOLLIP
P38 MAPK
MAPK3
PRKD1
PTGS2
Otud5
TRAF1
MYH11
p85 (pik3r)
PTPN11
PELI1
HERPUD1
SRC
TOM1
Akt
ARRB2
TRAF3
ATP2A3
NOS2
mir-135
PIK3R1
GFAP
p70 S6k
MAPK14
MYH10
MAP3K14
ICK
Rac
Ikk (family)
MPO
ARRB1
YES1
MAPK1
TUBB8
LYN
Mapk
IRAK
GPT
S100B
FAM89B
TRAF6
MAPK8
MTOR
NFKB1
MATR3
FOS
STAT1
EGR1
HMGB1
RELA
SRF
Tnf (family)

I

PIX - ↑ 10, ↓ 12

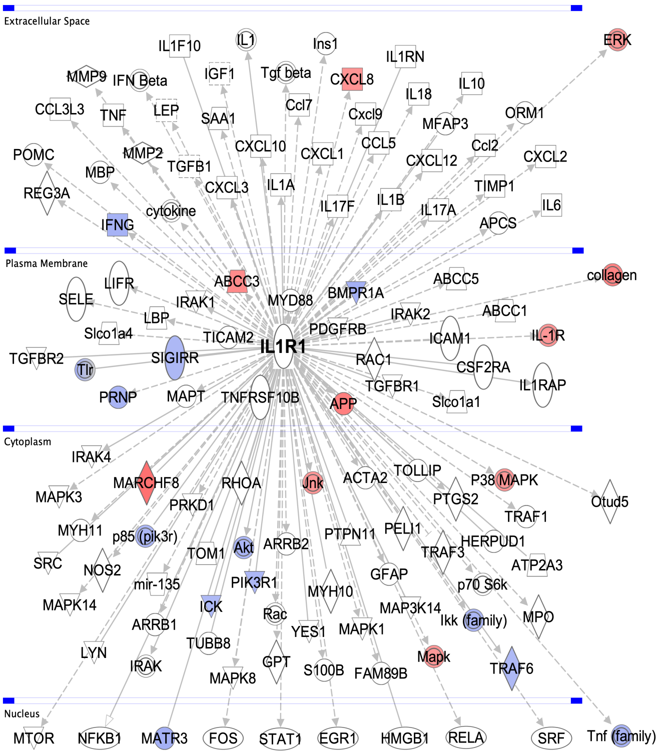

J

PX - ↑ 10, ↓ 5, ↑↓ 5

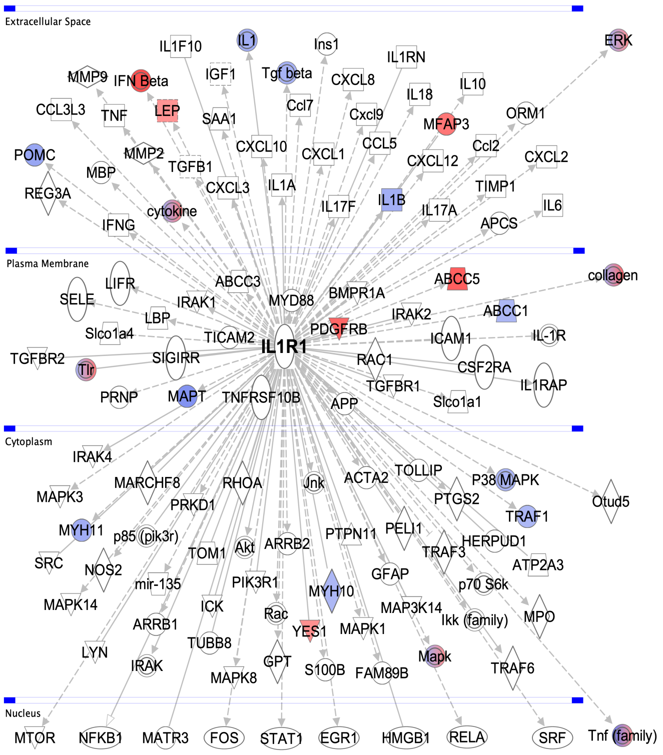

Supplementary Figure 2. Supplementary data for Figure 2

A, Heatmap comparing the post-treatment (7 days) to pre-treatment samples in individual patients (cut-off FC>1.5). **B-J**, Networks of of *IL1R1*-dependant genes, which were significantly regulated. The number of upregulated (red) or down-regulated (blue) genes is indicted by the arrows.

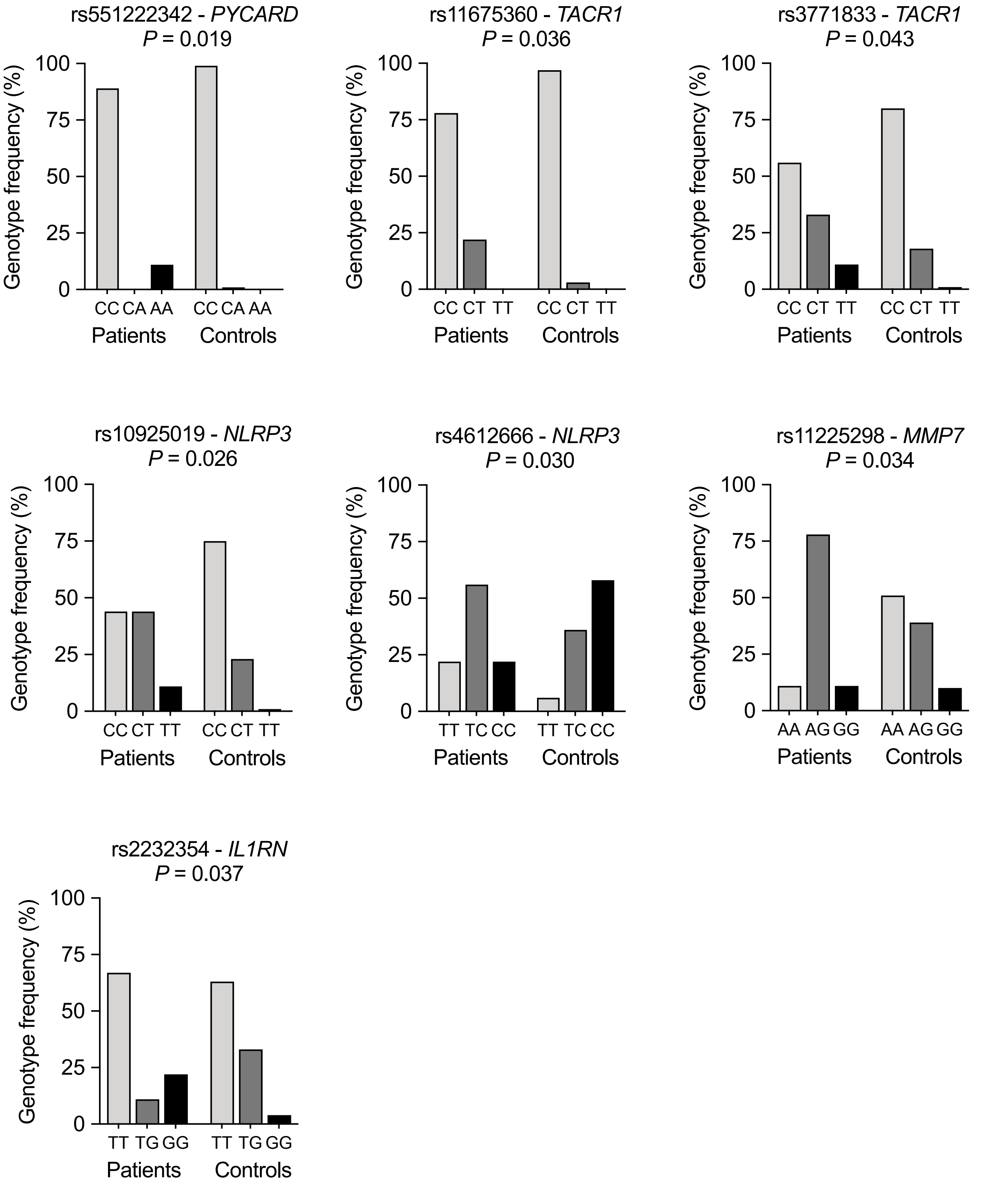

Supplementary Figure 3. Supplementary data for Figure 4.
Additional significant genetic variants in patients with bladder pain syndrome are shown. Histograms show genotype frequencies of disease associated SNPs compared to the 1000 Genomes control database (European population).

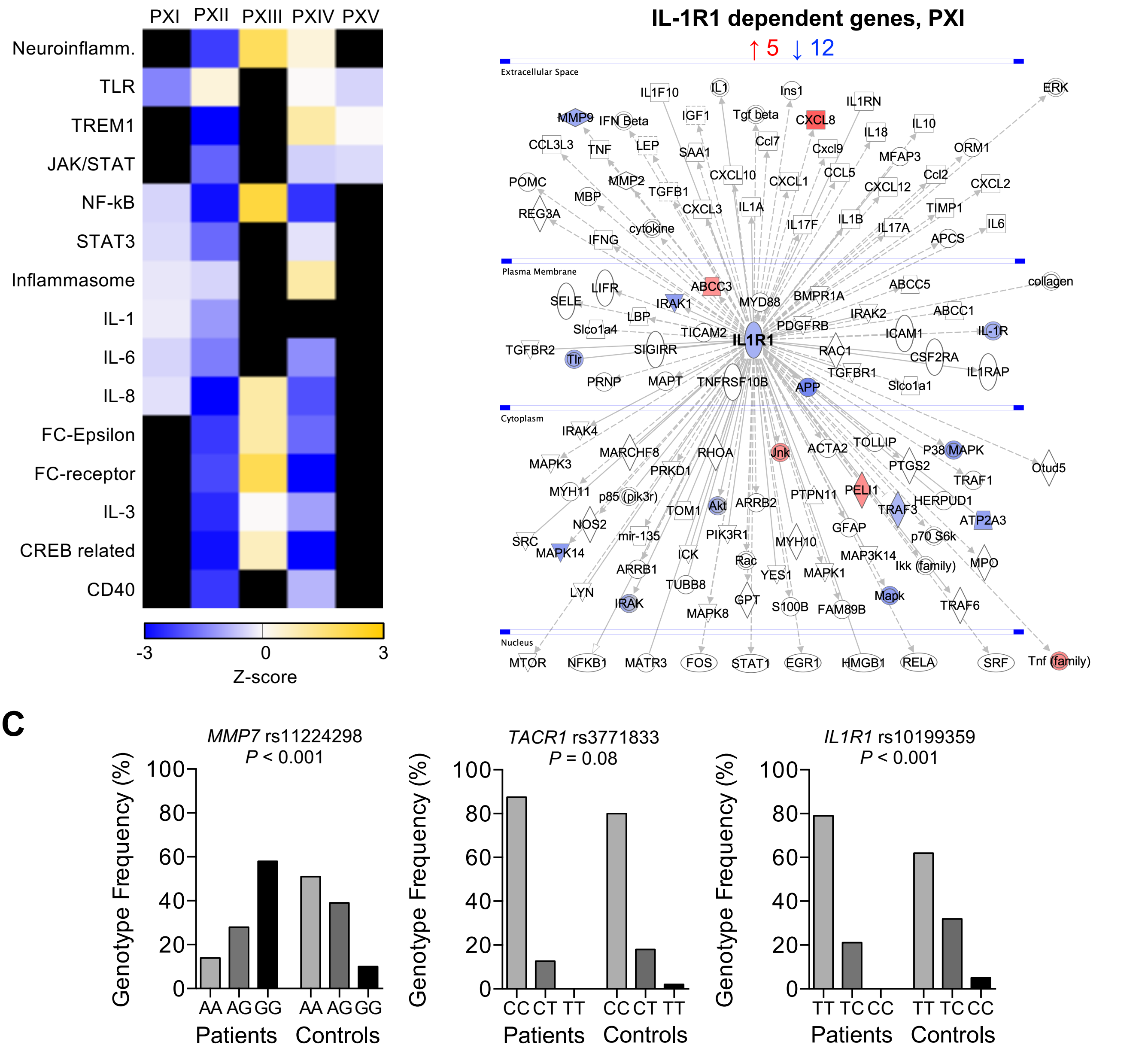

Gene	SNP ID	Alternative allele	Patient Genotypes							Allele Frequency %		*P*-value
										Patients	Control	
MMP7	rs11225298	G	AA	GG	GG	AG	GG	AG	GG	67	30	<0.001
TACR1	rs3771833	T	CC	CT	CC	CC	CC	CT	CC	28	11	0.002
IL1R1	rs10199359	C	TC	TT	TC	TT	TT	TC	TT	42	22	0.002

Supplementary Figure 4. Transcriptomic- and genetic analysis of validation controls
A, Gene expression was examined in five patients, using paired samples from day 0 and day 7. The expression of neuro-inflammatory- and IL-1-related genes was inhibited after seven days in responders from whom paired samples were available (n=3). Furthermore, inflammasome-, IL-6-, IL-8- and STAT3 pathway genes were inhibited. In one of the responders, neuroinflammation was weakly activated. No change was seen in the non-responder from whom paired samples were available. **B,** Pyrosequencing identified *IL1R1* and *MMP7* polymorphisms also in the second group of patients. *TACR1* or *IL1RN* variants were not detected.
